# Latest Advancements and Future Directions in Prostate Cancer Surgery: Reducing Invasiveness and Expanding Indications

**DOI:** 10.3390/cancers17183053

**Published:** 2025-09-18

**Authors:** Valerio Santarelli, Roberta Corvino, Giulio Bevilacqua, Stefano Salciccia, Giovanni Di Lascio, Francesco Del Giudice, Giovanni Battista Di Pierro, Giorgio Franco, Simone Crivellaro, Alessandro Sciarra

**Affiliations:** 1 Department of Maternal-Infant and Urological Sciences, “Sapienza” Rome University, Policlinico Umberto I Hospital, 00185 Rome, Italy; 2 Department of Urology, University of Illinois at Chicago, Chicago, IL 60607, USA

**Keywords:** prostate cancer, surgery, RARP, prostatectomy

## Abstract

For more than 20 years, after the introduction of the first robotic system, research on prostate cancer (PCa) surgery has mainly focused on evaluating outcomes of Robotic-Assisted Radical Prostatectomy (RARP). In the last few years, however, a new generation of innovative techniques, surgical approaches, and expanded indications have emerged. The Single Port (SP) robotic system was the first real hardware innovation in robotic surgery, and has already demonstrated advantages in terms of shorter length of stay, better cosmetic results and reduced postoperative pain. Artificial Intelligence (AI)-powered algorithms are being proposed as reliable tools for surgical assistance, aiding in standardization and mass implementation of robotic training. New surgical indications are emerging on the basis of patient and tumor characteristics. The extensive adoption of PCa screening and the precision of diagnostic tools have increased the rate of PCa diagnoses in a localized stage. Partial prostatectomy, despite needing further validation, has emerged as a safe and minimally invasive treatment option for confined tumors, able to minimize the side effects of prostate surgery. For locally advanced PCa, radioguided surgery has not only enhanced the oncological effectiveness of lymphadenectomy by enabling the precise identification and extraction of pathological lymph nodes, but has also contributed to minimizing the side effects associated with unnecessarily extensive dissections. Finally, in light of the increased efficacy of modern systemic therapies and the longer life expectancy, RP is currently being evaluated for primary tumor management in the metastatic phase. Despite the novelty of the aforementioned treatment options, they are already set to shape the future evolution of PCa management and international guidelines.

## Simple Summary

The advantages of a robotic approach to radical prostatectomy are well established. Currently, the surgical management of prostate cancer is benefiting from a new wave of technological innovations, such as the single port robotic platform and the applications of artificial intelligence. Moreover, the potential applicability of novel precise diagnostic tools in surgical practice is under investigation, to limit the extent of primary tumor resection (partial prostatectomy) and lymph node dissection (radioguided surgery). Finally, as the future of cancer treatment relies on multidisciplinary management, the role of surgical control of the primary tumor is being evaluated even in the metastatic setting, in combination with personalized systemic treatment. In this review, we aim to discuss the current status and future perspectives of the latest innovations that have the potential to shape the future of prostate cancer surgery.

## 1. Introduction

Prostate Cancer (PCa) is the most commonly diagnosed cancer in men worldwide and the second leading cause of malignancy-related death in American men, with a mortality of 18.7 per 100,000 men [1,2]. Currently, 70% of new PCa diagnoses are made at a localized stage, and 14% at a regional stage [3]. Since its first description in the late 19th century, Radical Prostatectomy (RP) has been the main surgical treatment option for localized and locally advanced PCa [4]. It involves the removal of the prostatic gland, seminal vesicles and distal vasa deferentia, followed by a vesicourethral anastomosis to restore continuity of the urinary tract and physiological voiding function [5]. Vascular damage, nerve injury, rectal injury, rectourethral fistula and lymphocele are the most common short and intermediate term complications of RP. Long-term functional complications, with major impact on patients’ quality of life (QoL), are urinary incontinence (UI) and erectile dysfunction (ED) [6]. While the rationale behind RP remained the same, novel technological advancements, surgical techniques and minimally invasive approaches helped reduce its mortality and morbidity, in terms of complication rates, length of stay (LOS) and functional results [7]. Currently, the Robotic-Assisted is the most performed approach to perform an RP worldwide. The advantages of minimally invasive approaches, mainly Robotic-Assisted Radical Prostatectomy (RARP) and Laparoscopic Radical Prostatectomy (LRP), over the open approach, are well established [8,9,10,11,12]. Despite available results from several clinical trials, proposing better outcomes of RARP over LRP, in terms of sexual potency recovery and the status of surgical margins (SMs), current guidelines still recommend informing candidates for RP that a clear superiority of either RARP or LRP over the other has not yet been demonstrated [10,13,14,15]. The choice should still rely on the surgeon’s confidence with one or the other approach and patients’ preference. During the years, RP has been successfully performed with several different accesses, primarily perineal, retropubic (either transperitoneal or extraperitoneal), transvesical, Retzius-sparing (transperitoneal or extraperitoneal), depending mainly on the technique of choice and surgeon’s preference, rather than patient’s characteristics [16,17]. While RP remains a standardized procedure, main variations include the approach of choice and the decision to perform a Pelvic Lymphadenectomy (PLND) or a Nerve Sparing (NS) procedure. An NS procedure is performed in well-motivated patients, after thorough discussion on the basis of preoperative sexual function and preoperative characteristics, with the aim of preserving erectile function [18]. According to current guidelines, the decision to perform a PLND should be made when validated predictive nomograms predict a ≥5% risk of nodal involvement, and in all high-risk cases [19,20]. The extended dissection, which involves the removal of obturator and external and internal iliac lymph nodes, is the template recommended by international guidelines [21].

For more than two decades, after the introduction of the first robotic system, research has focused on evaluation of RARP outcomes, alone or in comparison with those of ORC and LRP. However, in the past five years, a new wave of innovative techniques, approaches, and indications have emerged or are being proposed, with a view to transform the landscape of prostate surgery. These advancements are pushing the boundaries of minimally invasive surgery, while simultaneously expanding the therapeutic indications of surgical treatment. This review aims to explore recent and ongoing advancements that have the potential to revolutionize the treatment of prostate cancer.

## 2. Single Port Robotic-Assisted Radical Prostatectomy

The introduction of the Single Port (SP) robotic system is the main surgical innovation of the last seven years. After FDA approval in 2018, the SP robot reached the European market in 2024, and its popularity is growing across European robotic surgeons. The single incision, the 3DHD fully articulating scope and the double-jointed instruments are designed to work properly even in narrow spaces, allowing SP urological surgical procedures to be performed extraperitoneally. Moreover, thanks to the 360° rotation of the single arm, a standard supine position can be adopted for virtually every SP robotic urologic surgery. The single incision reduces postoperative pain and opioid use, the supine position minimizes anesthesiologic complications, and the extraperitoneal access decreases the risk of bowel injury and postoperative ileus. These advantages ultimately allow almost every SP procedure to be performed in an outpatient setting.

### 2.1. The Procedure

**Figure 1:**
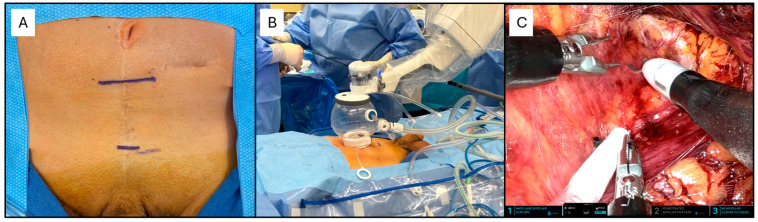
Set-up of SP RARP. (**A**) A single incision is made 4 cm above the pubic symphysis. (**B**) After insertion of the SP access port, and insufflation of the “fishbowl”, the robot is docked. (**C**) The intervention is carried out with Maryland bipolar forceps (Instrument arm #1), fenestrated bipolar forceps (Instrument arm #2) and monopolar scissors (Instrument arm #3).

The suprapubic extraperitoneal and the perineal approaches have been the standard during the era of ORC. With the advent of laparoscopic and robotic-assisted surgery, these approaches have been abandoned in favor of a transperitoneal access, which guarantees adequate space for multiple port insertion and reduces instruments’ conflicts. Nonetheless, thanks to its increased maneuverability and versatility, all of the aforementioned approaches have been successfully described with the SP robotic system [22,23,24,25]. The extraperitoneal suprapubic approach is currently the most commonly performed for SP RARP with and without PLND. The technique to perform an extraperitoneal SP RARP using the Da Vinci SP platform(Intuitive surgical Inc., Sunnyvale, CA, USA) has been described [26]. Briefly, access is gained through a single 3–4 cm incision 3–4 fingerbreadths above the pubic symphysis (Figure 1A). After clearance of the subcutaneous adipose tissue and incision of the linea alba, sufficient space is bluntly created to allow the insertion of the small SP access port. The valveless AirSeal^®^ (ConMed, Utica, NY, USA) is connected to an assistant port positioned in the dedicated space of the access port. After docking the robot (Figure 1B, the robotic instruments and the ROSI (Vascular Technology Incorporated, Nashua, New Hampshire, USA) suction irrigation system are inserted (Figure 1C). Subsequently, the procedure is carried out as per standard of Multi Port (MP) RARP.

### 2.2. Literature Synthesis

Consecutive retrospective series from expert robotic surgeons suggest that the more familiar transperitoneal suprapubic approach has been the approach of choice when engaging with SP RARP [22,27]. However, more recent data, mostly from the same centers, shows how after an initial adaptive phase all of the previously mentioned approaches can be successfully carried out with the SP robotic platform [28]. This pragmatic, step-by-step progression is a well-established method in surgical training, allowing one to concentrate on the innovative aspects of the technique, while keeping other variables constant. The theoretical advantages of an extraperitoneal approach are well accepted. Controversially, a recent systematic review by Jiang et al. comparing transperitoneal and extraperitoneal SP RARP found lower estimated blood loss (EBL), inferior complication rates and higher continence recovery of the former, while the latter demonstrated lower LOS (Table 1) [29]. However, it must be noted that, being that the majority of the primary operators experienced robotic surgeons, they were most likely more proficient with the transperitoneal approach. This discrepancy, which was actually reported in two of the five included studies, will probably be resolved in the future, as younger trainees are being exposed to different approaches. The most recent systematic review and meta-analysis comparing SP and MP RARP was published by Yuan et al. in 2024 (Table 1) [30]. Authors included both studies reporting outcomes of SP RARP alone and in comparison with MP RARP. For the SP RARP cohort, a total of 24 articles and 1385 patients were finally included. Results demonstrate a 21% positive SMs rate, a 93% rate of 12-month continence recovery and a 53% 3-month potency recovery rate. Regarding the comparative analysis, SP RARP and MP RARP showed similar results in terms of erectile function recovery, continence recovery and biochemical recurrence rates. SP RARP, however, showed significantly shorter LOS and better cosmetic results. Nonetheless, despite the relatively high number of included studies, it must be noted that they were mostly retrospective in nature and with a high level of heterogeneity. While available literature consistently suggests the safety and feasibility of SP RARP, concerns have been raised regarding the complexity of the procedure and a hypothesized longer learning curve (LC). So far, two studies have been published regarding the LC analysis of SP RARP, with conflicting results. The first one, evaluating the risk of postoperative complications according to the surgeon’s level of experience, found the minimum complication rate to be achieved only after 150 SP RARP cases [31]. More recently, however, a LC analysis adopting the cumulative summation (CUSUM) methodology of operation time found 42 and 20 to be the number of cases required to gain proficiency with SP RARP, respectively, with and without PLND [32]. In the future, prospective multicenter studies are required to better assess the potential benefits of the SP approach to RARP and to more precisely characterize the learning process needed to achieve them.

## 3. Role of Artificial Intelligence (AI) in Prostate Cancer Surgery

Artificial Intelligence (AI) has permeated every aspect of human life, and is being extensively used in healthcare with the aim of improving patients’ outcomes and optimizing healthcare workers’ time efficiency. Despite the first description of AI dating back to World War II, significant efforts of integrating AI in the medical domain have not been described until advances in recent years [33]. For instance, the first groundbreaking advancement in this direction has been the introduction of the CardioAI tool, adopting a deep learning algorithm to instantly decrypt cardiac MRI with excellent accuracy [33]. Radiology has probably been the field of medicine with the highest input of novel AI tools. Nonetheless, various companies have emerged in the field of medical AI, contributing to different aspects of healthcare, from data collection to treatment guidance and financial analysis [33,34,35]. In the surgical field, and particularly in robotic-assisted surgery, AI has been extensively used for segmentation of surgical procedures, critical procedural phases identification, feedback acquisition and anatomical structure recognition.

### 3.1. Key Surgical Steps Identification and Error Feedback

In 2024, Khanna et al. developed and proposed an AI-based computer vision algorithm able to automatically and independently identify and label surgical steps in RARP [36]. A total of 474 full-length RARP surgical videos were manually annotated under expert supervision to create a reference dataset. Of these, 292 were used to train the algorithm, 69 for internal validation, and 113 for testing its performance. The AI algorithm achieved an overall concordance rate of 92.8% compared to manual annotations, with its highest accuracy in identifying the vesicourethral anastomosis step (97.3%) and the lowest in the final inspection and extraction step (76.8%). The significance of these results lies in addressing one of the major barriers in the surgical training field: the absence of structured and labeled surgical video data. Adequately annotated surgical videos are extremely valuable not only for performance evaluation and feedback, but also for surgical training and education. Regarding skill assessment and error detection, Sirajudeen et al. in 2024 developed an AI model with the aim to estimate surgical skill and detect mistakes [37]. A total of 54 RARP videos from the SAR-RARP50 open dataset were blindly annotated at the gesture level using manual objective assessment tools and the Objective Clinical Human Reliability Analysis (OCHRA) method and used for training deep learning AI-powered models. AI-assigned skills scores were then compared with the human-assigned scores, showing moderate positive relationship with a deviation of 17.9–20.6%. While this AI model is not yet accurate enough for clinical use without human oversight, it provides a benchmark for future research.

### 3.2. Recognition of Relevant Anatomy

**Figure 2:**
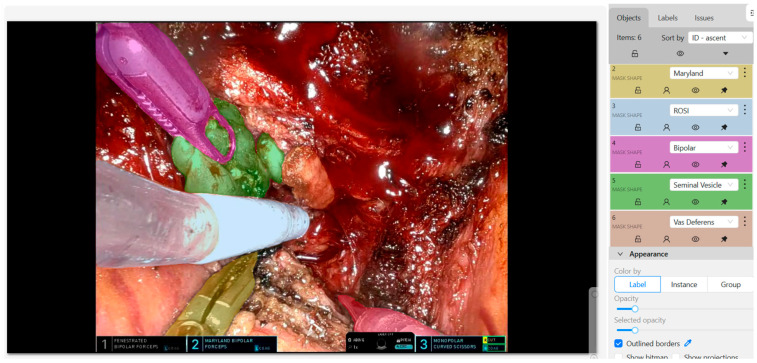
An example of how a screenshot from a surgical video can be annotated using specific software such as CVAT. Many annotated screenshots from different surgical videos can be used to train YOLO-based (or similar) AI models.

An AI model integrated in the surgical console, able to recognize with good accuracy and at an early stage relevant anatomical structures appearing in the surgical field of view could be extremely useful, especially during an initial training stage, to support decision making. Detection, segmentation, and classification are the three crucial steps in the development of such AI models. Detection involves the identification of surgical tools or anatomical landmarks using bounding boxes. Segmentation further delineates these objects with precise contours at the pixel level. Classification assigns meaningful semantic labels to the identified regions. This process often begins with high-quality image acquisition from surgical videos, followed by manual annotation using tools such as CVAT (Computer Vision Annotation Tool) [38] (Figure 2). Annotated datasets are used to train YOLO (You Only Look Once)-based models, which learn to predict bounding boxes and labels for objects in new, unseen images. These models are optimized through evolutionary algorithms and evaluated using metrics like mean Intersection over Union (mIoU), Dice Similarity Coefficient (DSC), and F1-score [39,40]. Similarly, a recent study explored the application of a convolutional neural networks (CNNs)-based deep learning model for semantic recognition in RARP [41]. Authors trained their model on 2400 images from 120 intraoperative videos, focusing on recognition of surgical instruments and anatomical structures. Dice scores for the identification of the instruments, bladder, prostate, and seminal vesicle-vas deferens were 0.96, 0.74, 0.85, and 0.84, respectively. However, CNNs inherently focus on local spatial features and lack the ability to interpret global contextual and temporal relationships within a scene. Integrating CNNs with Transformers (understanding object relationships) and Recurrent Neural Networks (RNNs), or Long Short-Term Memory (LSTM) (interpreting dynamic cues such as arterial pulsation or bowel peristalsis), could provide highly predictive and accurate feedback during robotic operations such as RARP [42,43].

### 3.3. Future Directions: Interdisciplinary/Interinstitutional Collaborations and Integration in the Robotic Console

Major limitations of currently developed AI models are overfitting, namely the tendency to perform well on training data but poorly on unseen data, and domain shift, which occurs when models trained in one setting lose accuracy when applied to different populations or clinical environments. Actually, results of previously discussed studies are mostly based on single center and single platform procedures, mostly achieved on retrospectively collected videos rather than on real-time surgical operations. In order to develop an efficient and up-to-date AI model, with sufficient accuracy to be integrated in clinical practice, interdisciplinary collaboration is fundamental. The practical medical knowledge and procedural expertise of clinicians should be evaluated through technical lenses of engineers and data scientists, to develop and maintain highly complex models. Furthermore, providing large and diverse datasets, thanks to multi institutional collaborations, is crucial to overcome the issue of overfitting and increase generalizability. AI features, mainly in the form of performance feedback and post-procedural analysis, are already being incorporated into novel robotic platforms [44]. In the future, AI models could be integrated into the surgical console, providing useful information, such as real-time feedback, alerts for the proximity of critical vasculature or suggestion of the optimal dissection plane. However, extensive validation through multicenter prospective real-time studies is required, alongside clear regulatory frameworks and ethical approval.

## 4. Partial Prostatectomy

RP, the main treatment option for localized PCa, is a standardized and effective procedure, with low mortality, complications, and recurrence rates. Nonetheless, rates of long-term urinary incontinence and erectile dysfunction after RP range between 4% and 40%, and 14% and 90%, respectively, depending on patients’ preoperative function, surgical approach and technique adopted [45,46,47]. The relatively high rates, paired with the high prevalence of PCa survivors, make these functional complications an unmet need with a significant burden on patients’ QoL and healthcare economics. Focal therapies, such as high-intensity focused ultrasound (HIFU), have emerged as an acceptable alternative in selected low- and intermediate-risk PCa patients with good life expectancy and good preoperative continence and erectile function [48,49]. However, even for well-selected patients, reported residual cancer and retreatment rates range between 25% and 81%, and 25% and 27% [50,51,52]. In this scenario, partial prostatectomy has been proposed as a safe and feasible treatment option, with similar advantages in terms of erectile function and continence sparing as focal treatment, but with the oncological effectiveness of a surgical removal of the tumor [52].

### 4.1. Anterior Prostatectomy and Precision Prostatectomy 

**Figure 3:**
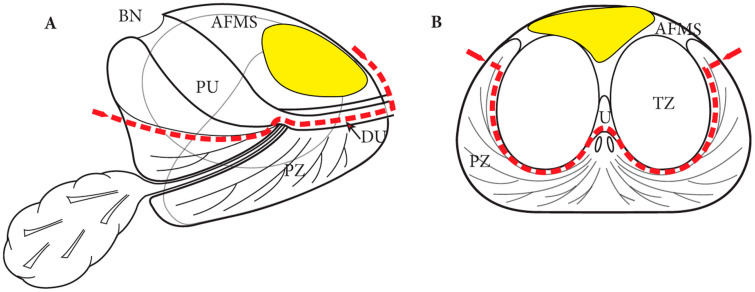
(**A**) Sagittal and (**B**) transverse schematic representation of the dissection plane of APP (red dotted line). An explanatory anterior PCa is depicted in yellow. BN: Bladder neck; PU: Prostatic urethra; AFMS: Anterior fibromuscolar stroma; PZ: Peripheral zone; DU: Distal urethra. (Reprinted with permission from Villers et al. [55], 2017, Inderbir S. Gill, Adil Ouzzane, Sebastien Crouzet, et al., license number: 6044941239010).

Anterior PCa, namely PCa located in the preboundary of the transition zone (TZ) and the anterior fibromuscular stroma (AFMS), accounts for approximately 3–5% of new PCa diagnoses [53]. For localized treatment of anterior PCa, focal therapies, including HIFU, are less effective compared to posterior PCa, and carry higher risks of external sphincter damage [53,54]. Robot-assisted anterior partial prostatectomy (APP) was first described by Viller et al. in 2017 [55]. Briefly, after prevesical space development without removal of adipose tissue covering the anterior surface of the prostate and bilateral endopelvic fascia incision, the dorsal venous complex (DVC) is secured and divided. The ablative part of the procedure involves removal of anterior half of urethra and apex, anterior bladder neck, median lobe, TZ, and anterolateral parts of the peripheral zone (PZ) (Figure 3). For reconstruction, the anterior bladder flap is advanced and sutured to the remaining half of the urethra and edges of PZ, to obtain a urethro-prostato-vesical anastomosis. Recently, the same authors published long term results of a series of 28 robotic-assisted APP. The 7-year post-APP freedom from cancer recurrence rate was 62.7% (35–81.3%), with salvage completion RP required for 8 patients [56]. These results suggest acceptable oncological outcomes with better functional outcomes when compared with RP.

Precision prostatectomy was hypothesized in 2019 by Sood et al. in a preclinical study, after analyzing PCa specimens from 100 RP patients whose tumor met the criteria for focal treatment [57]. Simulation analysis of the novel precision prostatectomy technique showed residual cancer rates of 14%, compared to 68% of focal therapy. The procedure, which involves removal of all prostatic tissue except a thin rim of tissue overlying the NVB contralateral to the dominant lesion, was described in detail by the same authors three years later [58]. Eighty-eight PCa patients with Prostate-Specific Antigen (PSA) ≤ 20 ng/mL, clinical stage ≤ cT2, a dominant unilateral lesion with Gleason Score (GS) ≤ 7 (4 + 3), no contralateral Gleason ≥ 4 lesion and good preoperative sexual function were included. At 12 months, 100% and 85% of patients were completely continent and potent, respectively. At 36 months, 91.7% of patients did not require additional treatment and all patients were alive and free of metastatic disease.

### 4.2. Single Port Transvesical Partial Prostatectomy

The advantages of SP RARP, in terms of lower invasiveness, shorter LOS and better cosmetic results, have already been described. SP Transvesical SP RARP is a complex procedure, but safe and effective especially for confined lesions and small prostates, with reported faster continence recovery when compared to the standard retropubic approach [59]. The first series of SP transvesical Robot-assisted partial prostatectomy (SP-TVRAPP) was published by Kaouk et al. in 2022 [60]. Inclusion criteria followed those adopted for HIFU (GS ≤ 7, no evidence of locally advanced or metastatic disease at preoperative MRI), but were expanded to include anterior tumors, significant calcifications and larger prostates. Real-time tumor and urethra localization is achieved by intraoperative fusion of live transrectal ultrasound images and preoperative MRI. In this study, 2 anterior and 7 lateral (5 left and 2 right) PCa patients were included. Monolateral posterior dissection, including dissection of seminal vesicle, vas deferens and NVB is performed for laterally located tumors, but not for anterior lesions. Similarly, ipsilateral vascular pedicle dissection is required for lateral gland excision. The anterior bladder neck is incised at the 10 o’clock position for left tumors, 2 o’clock position for right tumors, and bilaterally for anterior lesions. For anterior tumors, only prostatic tissue anterior to the urethra is removed. For lateral lesions, a lateral gland excision, following the urethra at the midline, is performed. Intraoperative frozen section examination of the margins of the remnant prostatic surgical area is carried out before proceeding with vesicourethral anastomosis. Despite the negative intraoperative frozen sections, four patients had positive focal margins at final pathological analysis. Regarding functional outcomes, potency and urinary continence were recovered in all patients within the 6th postoperative week. Long-term results of SP-TVRAPP were reported on a cohort of 20 PCa patients by Pedraza et al. in 2025 [61]. A majority of 93.7% of patients recovered complete continence and potency (defined as having erections sufficient for penetration) at 12 months post procedure. Overall, 85% of patients had negative surgical margins at final pathological analysis. After a mean follow-up of 15.5 months (0.2–34.8), no patient required secondary interventions and all remained free of clinically significant or metastatic PCa.

### 4.3. Future Prospectives

The aim of partial prostatectomy is to preserve organ function while ensuring cancer is not inadequately treated. While it represents a significant breakthrough in treating PCa, additional research is crucial to identify those patients who would benefit from intensive surveillance rather than immediate aggressive treatments. Furthermore, more comprehensive long-term follow-up data from well-designed randomized controlled trials are required to precisely delineate cancer recurrence patterns, in order to pinpoint the definitive therapeutic niche of partial prostatectomy within the current armamentarium for localized PCa management. Effective monitoring strategies must be developed before definite validation, combining multiple diagnostic approaches, including PSA testing, precise imaging and both random and targeted biopsies.

## 5. Radioguided Surgery (RGS)

A key limitation of RARP is the lack of real-time intraoperative visualization of tumor extent and lymph node metastases. Negative surgical margins and lymph node metastases dissection during ePLND are essential factors in long-term cancer control and biochemical recurrence rates. Preoperative imaging and an accurate lymph node staging remains critical. Nowadays, intraoperative imaging-guided surgery has developed, aiming for a more precise, effective and patient-centered surgical approach [62].

### 5.1. Sentinel Node Biopsy (SNB) for Primary Staging

The first attempt of real-time intraoperative guidance is SNB. This technique aims to identify the first lymph node receiving drainage from the prostate tumor by injecting tracers into the peripheral gland zone (i.e., indocyanine green (ICG), radioactive agents, or hybrid fluorescent–radioactive compounds) [62].

Mazzone et al. evaluated SNB’s added value to ePLND in 1168 patients undergoing radical prostatectomy finding higher detection rates of positive nodes with SNB: the incidence of pN1 patients was 19% with ePLND alone, 28% with SNB plus a hybrid tracer, and 36% with SNB using ICG alone [63]. The hybrid tracer emerged as an independent predictor of lymph nodal metastases (LNMs) (OR 1.61; *p* = 0.002).

Lannes et al. conducted a multicenter study on 162 cN0M0 patients using a gamma probe following preoperative intraprostatic injection of 99mTc-nanocolloid [64]. SNB showed high diagnostic performance (95.4% sensitivity, 100% specificity, 100% PPV, and 99% NPV) as it identified LNMs in 14% of patients among successfully sentinel lymph nodes individuations (88%).

Despite promising results, SNB has some limitations. Micro-metastases, skip metastases and individual variability in the lymphatic drainage system may lead to underestimation of disease burden [62].

### 5.2. PSMA-RGS

PSMA-RGS exploits radiolabeled PSMA ligands to intraoperatively identify metastatic lymph nodes, potentially reducing unnecessary ePLNDs and improving lesion removal in salvage lymph node dissection (sLND). This method provides real-time feedback to the surgeon as the gamma-ray detection directly locates PSMA-avid tissue.

**Figure 4:**
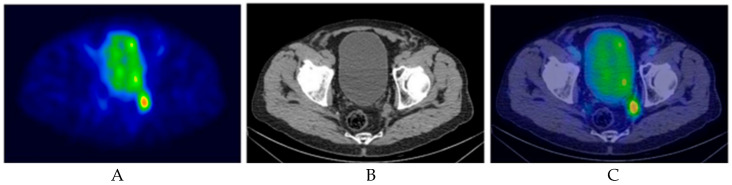
[99mTc]Tc-PSMA-I&S SPECT/CT. (**A**) Identification of a suspicious lymph node at the level of the left obturatory region, as shown in SPECT, (**B**) CT and (**C**) SPECT/CT—axial view (Reprinted with permission from Quarta et al. [66], 2024, Creative Commons CC BY).

Among the available radiotracers, [99mTc]Tc-PSMA-I&S (Figure 4) is the most widely used due to its cost-effectiveness, availability, strong in vivo stability, and high uptake in cancer lesions: it is administered 20 hours before surgery, then allowing preoperative SPECT/CT imaging as quality control [65]. During robot-assisted surgery, the drop-in gamma probe.

**Figure 5:**
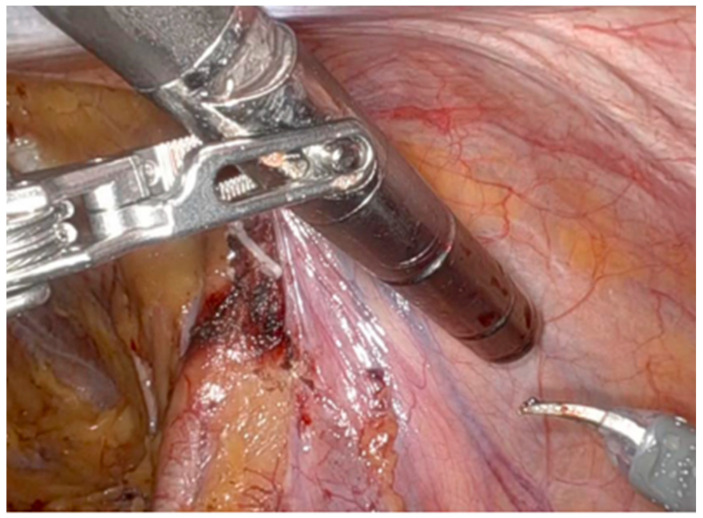
Drop-in gamma probe (Crystal Drop-In Probe; Crystal Photonics, Berlin, Germany). RGS aims to improve the surgeon’s ability to intraoperatively identify and remove LNMs and detect nodal micrometastases (<5 mm in size) that might be missed by preoperative imaging (Reprinted with permission from Quarta et al. [66], 2024, Creative Commons CC BY).

Figure 5 is introduced via a 15 mm assistant port above the right iliac crest, and it is controlled by the console surgeon. The probe’s control unit provides acoustic and numerical signals related to radioligand activity, leading to precise lymph node dissection. Typically, a positive signal is defined as a target-to-background ratio at least double that of adjacent fatty tissue. Furthermore, ex vivo gamma measurements may confirm lesion removal.

#### 5.2.1. Recurrent Prostate Cancer Setting

In PCa patients with biochemical recurrence (BCR) with suspected LNMs on PSMA PET imaging, sLND has emerged as a potential treatment option [67]. Traditional approaches to sLND have shown limited long-term cancer control: Bravi et al. reported a 10-year cancer-specific survival rate of only 66%, with many patients demonstrating persistently elevated PSA postoperatively, likely due to incomplete resection of metastases and limitations of conventional imaging [68].

Further needs to assess correctly the disease spreading and the intraoperative localization of LNMs have led to PSMA-RGS developing, by enhancing intraoperative visualization and targeting of PSMA-avid tissues [69].

Maurer et al. reported diagnostic accuracy (93%), sensitivity (84%), specificity (100%), PPV (100%), and NPV (89%) of this approach; moreover, a PSA reduction to <0.2 ng/mL was widely achieved, and a subset remained BCR-free during short-term follow-up [70].

De Barros et al. conducted the first prospective feasibility study of a drop-in gamma probe in 20 patients with up to three pelvic PSMA-PET-positive recurrences, resulting in 90% of lesion identification; false-negative LNMs were all < 3 mm, according to technical limitations in detecting micrometastases [71].

The largest cohort of patients undergoing PSMA-RGS sLND was described by Knipper et al. (*n* = 553), showing that 94% of patients (*n* = 522) had metastatic tissue removed. Treatment-free survival (TFS) rates at 2 years were 81%, 56%, and 43% (*p* < 0.001) for patients with PSA levels < 0.1, 0.1–0.2, and >0.2 ng/mL, respectively [72].

Moreover, Berrens et al. demonstrated a strong relation between PSMA-PET-SUVmax values ≥ 6 and intraoperative gamma count rates, suggesting its utility in optimizing patient selection for PSMA-RGS sLND [73].

#### 5.2.2. Nodal Staging During RARP with ePLND Setting

The first description of PSMA-RGS technique for PCa patients undergoing RARP with ePLND was made by Gondoputro et al.; their study aimed to evaluate intraoperative drop-in gamma probe signals compared with regional pathological results (Table 2) [74]. PSMA-RGS identified 32 positive nodal regions, 16 of which were confirmed as harboring metastases, resulting in 76% sensitivity and 69% specificity, with a NPV of 88%. Thereafter, Gandaglia et al. conducted a prospective study, demonstrating the probe sensitivity, specificity, PPV, and NPV of 67%, 100%, 100%, and 90%, respectively, at per-patient analysis [75].

The diagnostic accuracy of PSMA-RGS was supported by Yilmaz et al., reporting 100% of sensitivity, specificity, NPV and PPV on a per-node basis in 15 PCa patients who underwent RARP with ePLND [76]. Additionally, Schilham et al. evaluated PSMA-RGS in 20 patients with PSMA PET-positive lymph nodes, successfully removing 88% of the targeted lesions: 29 (59%) of the 49 lesions identified on imaging were detected intraoperatively, and 28 of these were confirmed pathologically [77].

Even though PSMA-RGS demonstrated feasibility and promising outcomes, the limited spatial resolution of the probe seemed to influence in vivo accuracy, as nodal metastases < 3 mm were missed in Gondoputro et al., Gandaglia et al. and Schilham et al. studies [74,75,77].

To further explore this concern, Quarta et al. investigated optimal tumor-to-background (TtB) ratio thresholds (≥2 vs. ≥3 vs. ≥4) in a prospective study comparing intraoperative findings with PSMA PET imaging, recommending a TtB threshold assessment of ≥2 to optimize sensitivity and minimize the risk of missing true positive lesions [66].

Nowadays, the impact of PSMA-RGS on long-term oncological outcomes remains uncertain and further evaluations are needed.

### 5.3. Fluorescence Guided Surgery (FGS) Using PSMA-Based Tracers

Recently, PSMA-targeted fluorescent tracers such as OTL78 and IS-002 have emerged as promising tools in FGS in patients undergoing RARP with ePLND due to their capacity to real-time intraoperative identification of PCa cells [78].

A phase II trial assessed how in 18 PCa patients, intravenous administration of 0.03 mg/kg OTL78 24 h prior to surgery yielded optimal signal-to-background ratio and tumor visualization both in vivo and ex vivo, without any severe adverse event [79]. The strongest diagnostic outcomes were observed in patients with high PSMA expression, larger tumor volumes, and ISUP grade group ≥ 3. Insufficient wash-out led to false-positives.

A phase I trial evaluated IS-002 in 24 high-risk PCa patients undergoing RARP with ePLND, resulting in high NPV for locoregional disease (100%) and LNMs (97%) with an optimal dose of 25 µg/kg [80]. Higher doses led to increased false-positives, and the most common effect observed was temporary urine discoloration due to renal clearance of the tracer.

The main limitations of PSMA-based FGS are the difficult identification of the involved nodes, since most of them are normal in size, and the limited utility of this modality in micrometastatic disease [80]. While IS-002, with its higher spatial resolution, may effectively overcome the latter limitation, results of future prospective phase II and multicenter studies are required to confirm the exploratory results currently available.

## 6. Role of Targeting Primary Tumor in Oligometastatic PCa

Oligometastatic PC (OMPC) represents an intermediate state of PCa in the progression from localized to widespread metastasis, of which radiographically significant sites are limited in number (3–5) and location. OMPC was traditionally treated with systemic therapy. Due to its favorable biology and more indolent natural history, OMPC has recently been studied to assess optimal imaging methods and treatment outcomes [81]. Particularly, the longer survival granted by novel systemic agents and the more precise disease characterization achieved with advanced molecular imaging has allowed research to focus on the integration of metastasis-directed therapy (MDT) and radiotherapy (RT) of the primary tumor into the therapeutic armamentarium for OMPC. More recently, evidence is emerging for the role of radical prostatectomy as cytoreductive treatment, particularly given the reduced morbidity associated with robotic-assisted surgery.

### 6.1. Current Standard of Care (SOC) and Based on Treatment Strategies

#### 6.1.1. Systemic Therapy

The current American Society of Clinical Oncology (ASCO) guidelines recommend doublet or triplet systemic therapy with ADT combined with agents such as docetaxel, abiraterone, enzalutamide, apalutamide, or darolutamide as SOC for mHSPC. However, there is still not an optimal patient selection and treatment sequencing definition [82]. Evidence from the CHAARTED trial suggests that docetaxel provides the most benefit in high disease burden, whereas the STAMPEDE trial assesses survival benefit from triplet therapy irrespective of burden tumor [83]. Furthermore, results from the ARASENS trial showed improved overall survival and time to castration resistance with ADT + docetaxel + darolutamide, with the pending burden of tumor subgroup data.

#### 6.1.2. Cytoreduction

In synchronous OMPC several promising results have emerged regarding the combination of systemic treatment and prostate radiotherapy (RT) cytoreduction.

Improved OS in low metastatic burden patients receiving prostate RT was observed in the STAMPEDE trial (81% vs. 73% OS at 3 years) [84]; benefits in time to PSA progression were subsequently seen in the HORRAD trial [85]; eventually, the best radiographic PFS was reported in the PEACE-1 trial, supporting the potential benefit of intensified multimodal therapy [86].

Traditionally employed for palliation, metastasis-directed therapy (MDT) is increasingly investigated for its capacity to delay tumor progression and potentially extend survival in OMPC patients through surgical resection or stereotactic body radiotherapy (SBRT) targeting metastatic sites. ESTRO clinical guidelines now recommend MDT in selected oligometastatic settings [87].

Several studies support the efficacy of MDT. A meta-analysis by Ost et al. showed that more than half of the patients remained progression-free 1–3 years following MDT [88]. Additional studies have demonstrated that SBRT, particularly in oligo-recurrent or nodal-only disease, can safely prolong PFS [89]. Enhanced precision in MDT delivery can be achieved through PSMA PET/CT imaging—notably 68GaGa-PSMA-11—which enables accurate identification of treatable metastases [90].

The OLIGOPELVIS GETUG P07 phase I trial focused on men with pelvic node-only relapse (six or fewer metastatic lymph nodes in the pelvic region): 67 patients received high-dose, intensity-modulated salvage radiotherapy directed at the pelvic nodes, alongside a six-month of ADT, resulting in a median PFS of 45.3 months and a median biochemical RFS of 25.9 months. To date, approximately 46% of the patients remained relapse-free three years after treatment, suggesting that localized MDT combined with a limited course of ADT can offer durable disease control in select patients with nodal recurrence [91].

These findings collectively suggest that combination strategies could represent an important step forward in the treatment of OMPC, particularly as patient selection criteria are refined through molecular and immunologic biomarkers.

### 6.2. Radical Prostatectomy in OMPC

Primary tumor cytoreduction through surgical or radiotherapeutic approaches represents a key investigational strategy in OMPC management. Multiple studies have demonstrated that reducing primary tumor burden can decrease circulating and disseminated tumor cell populations with metastatic potential, while enhancing immune responses through increased T helper to regulatory T cell ratios [92].

The SOLAR trial combined systemic therapy (leuprolide, apalutamide, and abiraterone) plus MDT via SBRT, and primary tumor treatment with either RP or RT. At six months post-treatment, 83% of patients achieved the primary PSA endpoint, with all patients remaining progression-free at a median follow-up of 31 months, suggesting the potentiality around this strategy [93].

In men with low-volume metastatic disease, cytoreductive prostatectomy (CRP) has emerged as a potential treatment option. Large retrospective analyses have found significant survival benefits in patients undergoing RP or prostate brachytherapy compared to those receiving no local treatment, with acceptable safety profile [94,95]. In a retrospective analysis, Heidenreich et al. reported a five-year OS rate of approximately 80%, and defined the use of neoadjuvant ADT, low metastatic burden, and favorable PSA levels as predictors for improved outcomes and reduced complication rates [96].

Several prospective clinical trials are currently underway. To date, the Italian multicenter experimental trial APPROACH is an ongoing study evaluating whether treatment with apalutamide in combination with ADT for 6 months followed by locoregional treatment with RT or RP has better efficacy than medical treatment with apalutamide plus ADT alone in terms of radiographic PFS in OMPC hormone sensitive patients [97].

Published and ongoing research is yielding encouraging results, paving the way for the establishment of novel therapeutic approaches for OMPC management, optimizing PFS rates and clinical outcomes. However, further evaluations are needed as many trials are still ongoing.

## 7. Conclusions

In the current review, we explored the latest advancements and future prospects of prostate cancer surgery. As non-surgical therapeutic options increased, they were thought to substitute and limit surgical indications. However, the novel techniques and approaches reported in this review largely debunk this theory. On the contrary, thanks to the minimal invasiveness of robotic (and particularly Single Port) surgery and the increased precision and effectiveness of radioguided surgery, surgical indications are expanding to even older and frailer patients with higher disease burdens, without compromising their safety. While the theoretic advantages of the aforementioned techniques are becoming increasingly clear, their widespread diffusion is currently limited by the high costs and lack of definite evidence. The SP system has already gained approval for the European market, but in order to increase its attractiveness, large multi-center cost analyses are required to justify the high costs of the platform and its disposables with the advantages of fewer complications and shorter hospital stays. Nonetheless, after only one year since its approval, the distribution of the SP platform and consequently the number of SP RARP performed is already increasing exponentially across Europe. Regarding partial prostatectomy and radioguided surgery, high-quality and long-term evidence is still needed before their introduction into routine clinical practice. Similarly, the mounting evidence regarding the applications of artificial intelligence in surgery will inevitably collide with the slower pace of regulatory and bureaucratic processes. Finally, in the last few years, due to the complex and rapidly expanding therapeutic arsenal, it has become clear that the future of prostate cancer treatment is multidisciplinary and personalized.

## Figures and Tables

**Table 1:** Most recent comparative systematic reviews of (A) transperitoneal vs. extraperitoneal SP RARP and (B) SP RARP vs. MP RARP SP RARP: Single Port Robotic-Assisted Radical Prostatectomy; EBL: Estimated Blood Loss; LOS: Length of Stay; PSM: Positive Surgical Margins; MP RARP: Multi-Port Robotic-Assisted Radical Prostatectomy; BCR: Biochemical Recurrence; ED: Erectile Dysfunction.

Author and Year	Intervention	Comparator	Included Studies	Outcomes
**A. Jiang et al., 2023 [29]**	Extraperitoneal SP RARP	Transperitoneal SP RARP	5 studies and 883 patients	**Intervention:** Higher EBL, complication rates and lower continence recovery; Shorter LOS.
**B. Yuan et al., 2024 [30]**	SP RARP	/	24 studies and 1385 patients	**Intervention:** 21% PSM; 93% 12-month continence recovery; 53% 3-month potency recovery.
	SP RARP	MP RARP	11 studies and 1553 patients	**Intervention**: Better cosmetic results and shorter LOS; No differences in PSM, BCR, incontinence and ED rates.

**Table 2:** **PSMA-RGS in primary PCa staging setting:** main studies regarding its possible application (Readapted from Quarta et al. [62], 2025, Creative Commons Attribution). HR-PCa: High Risk Prostate Cancer; LNI: Lymph Node Involvement; PSMA-RGS: Prostate-Specific Membrane Antigen Radioguided Surgery; LNMs: Lymph Node Metastases; RARP: Robotic-Assisted Radical Prostatectomy; ePLND: extended Pelvic Lymph Node Dissection; PPV: Positive Predictive Value; NPV: Negative Predictive Value; IR: Intermediate Risk; HRcN0cM0: High Risk clinically Node negative clinically Metastasis negative; PCa: Prostate Cancer; CIM: Critical Intermediate; PET: Positron Emission Tomography; [99mTc]TcPSMA-I&S: Technetium-99m PSMA Imaging and Surgery; [111In]InPSMA-I&T: Indium-111 PSMA Imaging and Therapy.

Author and Year	Cohort of Patients	Tracer Used	Main Goals	Outcomes
**Gondoputro** **et al.,** 2022, [74] prospective	12 HR-PCa patients with a LNI risk > 10%	[99mTc]TcPSMA-I&S	evaluating the safety and feasibility of PSMA-RGS to guide the intraoperative detection of LNMs during RARP with ePLND	per-lesion analysis: - sensitivity: 76% - specificity: 69% - PPV: 50% - NPV: 88%
**Gandaglia** **et al.,** 2022, [75] prospective	12 IR- or HRcN0cM0 PCa patients at CIM with a LNI risk > 5%	[99mTc]TcPSMA-I&S	reporting the planned interim analyses of a phase 2 prospective study aimed at describing PSMA-RGS during RARP with ePLND	per-lesion analysis: - sensitivity: 50% - specificity: 99% - PPV: 80% - NPV: 96%
**Schilham** **et al.,** 2024, [77] prospective	20 PCa patients with positive preoperative PSMA PET	[111In]InPSMA-I&T	evaluating the safety and feasibility of PSMA-RGS during RARP with ePLND	per-lesion analysis: - sensitivity: 66.7% - specificity: 99.8% - PPV: 96.8% - NPV: 97%
